# Ethical considerations in the regulation and use of herbal medicines in the European Union

**DOI:** 10.3389/fmedt.2024.1358956

**Published:** 2024-06-14

**Authors:** Anthony Raphael Gatt, Patricia Vella Bonanno, Raymond Zammit

**Affiliations:** ^1^ Department of Moral Theology, University of Malta, Msida, Malta; ^2^ Department of Health Systems Management and Leadership, Faculty of Health Sciences, University of Malta, Msida, Malta; ^3^ Strathclyde Institute of Pharmacy and Biomedical Sciences, University of Strathclyde, Glasgow, United Kingdom

**Keywords:** herbal medicines, traditional medicine, pharmaceutical regulation, access to healthcare products, pharmacovigilance, moral awareness

## Abstract

The regulation and use of herbal medicines is a topic of debate due to concerns about their quality, safety, and efficacy. EU Directive 2004/24/EC on Herbal Medicinal Products was a significant step towards establishing a regulatory framework for herbal medicinal products in the EU, and bridging the gap between conventional and herbal medicines. This Directive allows herbal medicinal products to be marketed in the EU through full marketing authorisation, well-established use, and traditional use of herbal medicinal products. The framework relies on the correlation between the therapeutic claims of herbal medicine and the scientific evidence backing them up: the greater the claims made regarding medicinal benefits, the more evidence is required to substantiate its efficacy and safety. This regulatory framework acknowledges and incorporates traditional knowledge when evaluating herbal medicines, showcasing a balanced approach that values cultural traditions while mandating monographs for traditional herbal medicinal products. Excluding herbal medicines completely limits access to affordable treatment, particularly when they serve as the only alternative for some, and protects consumer autonomy. This EU framework could therefore serve as a practical guidance for the use and regulation of herbal medicines, even outside the EU. In conclusion, it is argued that the same moral imagination and courage shown by regulators in the case of herbal medicines could perhaps be used in the regulatory frameworks of other healthcare products.

## 1 Introduction

Many people assume that natural products are always safe. This misconception leads to a high usage of herbal products in the EU. Unfortunately, this increase has also led to a significant rise in the number of reported adverse reactions caused by these products (1). As a result, herbal products are now being regulated to address these quality, safety, and efficacy concerns.

The regulation of herbal medicines is a complex issue that poses various ethical challenges. These challenges arise due to the conflicting philosophical approaches between Western conventional medicine and traditional herbal medicine, contrasting claims of multiple stakeholders involved in the herbal medicine market, conflicts between duty-based and consequentialist ethics, the lack of relevant information and guidance, insufficient substantiating proof, and cultural differences that clash with ethical principles. These dilemmas may fall into moral “grey areas”, necessitate the balancing of short-term and long-term consequences, bring public scrutiny, and may present conflicts between ethical principles. Moreover, these dilemmas present themselves in situations involving strong, inherent intuitions and emotions which may lead to biases. All these issues made it difficult for regulatory authorities to regulate the sector.

The European Union (EU) has a responsibility to acknowledge and respect the diverse cultures and financial abilities of its citizens. It should consider the viewpoints and interests of all stakeholders and weigh conflicting ethical principles, such as autonomy and beneficence, as well as prioritize specific values depending on the context and potential impact. Regulators must therefore make decisions in a transparent manner and be accountable for their actions. With more and more people in the EU turning to herbal medicines, it is essential that the EU health authorities continue to regulate and protect the primary interests of patients and customers.

Precise figures for the global usage of herbal medicines, both regulated and unregulated, are difficult to calculate because the demand differs significantly from region to region due to socioeconomic and cultural factors. However, present pharmaceutical sales data prove a significant rebirth of interest in herbal medicines in all developed countries. Between 2024 and 2029, the European herbal medicine market is projected to develop at a compound annual growth rate of 6.02%, with a projected market size of USD 65.3 billion in 2024 and USD 89.05 billion in 2029 (2).

Several reasons might explain the rapid expansion in the use of herbal medicines, including the proliferation of chronic diseases (2), increased access to information, the various “claims” on their effectiveness, the improvement in quality of these products due to advances in technology, an increased environmental awareness leading to preferences for natural treatments, and the perception that herbal medicines are safe (3). This increased public interest also arises from drug-related issues. These include the high cost of certain conventional medications, fear of adverse reactions from synthetic drugs, the increased perception that herbal medicines are superior to manufactured drugs, the trend to move from synthetic to organic medicine and the lack of effectiveness of Western medicine on chronic ailments (4).

This rise in the popularity of herbal medicines is also due to increased self-medication by customers (5). Patients today want to be informed to be empowered to make their own choices, and thus to be able to determine their own methods for well-being. They are not happy to narrate their private concerns to physicians or pharmacists because they fear a lack of confidentiality in handling their health information (6). Many opt for herbal medicines based on a deductive approach based on anecdotal information (4). Persons are increasingly motivated to accept the therapeutic value of a treatment based on personal beliefs or intuition rather than scientific reasoning (4). The media also readily supplies sensational news about herbal medicines.

The herbal medicine industry recognizes the growing popularity of herbal medicine and aims to capitalize on this trend to boost its profits. To achieve this, many herbal medicine manufacturers employ effective marketing strategies to promote their products to the public. Some invest millions of dollars in advertising to establish their product's credibility and earn the trust of consumers. These advertisements appeal to consumers of all ages, enticing them with a range of benefits, such as aiding the growth and mental development of children, enhancing euphoric experiences for young adults, managing daily stress, slowing down the aging process, and improving memory and cognitive function. Through these innovative products and targeted marketing campaigns, the herbal medicine industry is poised to deliver products that meet the diverse needs of consumers while also maximizing their returns on investment.

The increased use of herbal medicines by consumers, however, has had its negative consequences. Consumers and physicians have reported several adverse reactions and herb-drug interactions (7), some of which were serious and even resulted in death (8). After these reports, it became clear that the quality, safety and efficacy of herbal medicines needed to be regulated to safeguard patients.

This argumentative review thus follows this trajectory: first, the main operational terms will be defined, in particular, that this paper deals with herbal medicinal products and excludes non-medicinal herbal products, such as food supplements or cosmetics, which are considered outside the scope of this review; then, the EU regulatory framework is described; following which the ethical issues relating to the authorisation and use of herbal medicinal products will be identified. Then, the views of various stakeholders in the herbal medicinal market will be presented, followed by a discussion and a conclusion.

## 2 Classification and definitions of herbal products

In the EU, herbal products that are intended for use as dietary supplements are regulated under Regulation (EC) No. 178/2002 (9) and Directive 2002/46/EC (10), whereas cosmetic products, including herbal preparations for cosmetics, are governed under Regulation (EC) No. 1223/2009 (11) which repealed EU Directive 76/768/EEC (12). This review, however, focuses on herbal medicines. It, therefore, excludes any concern related to dietary supplements and cosmetics which are totally outside the scope of this review. The difference in products and their regulatory regime is summarised in Table 1.

**Table 1:** Classification of different herbal products and their respective regulatory status.

Herbal products (any product made from herbs or plants, with or without authorisation)			
Herbal food	Herbal cosmetics	Herbal medicines	
Regulated by	Regulated by	Regulated by	Unregulated
Regulation (EC) No. 178/2002 and Directive 2002/46/EC	Regulation (EC) No. 1223/2009	EU Directive 2001/83/EC as amended by EU Directive 2004/24/EC	** **
		Herbal medicinal products: regulated and authorised. These can be authorised: (1) traditional herbal medicinal products; (2) well-established use; and (3) marketing authorisation as a medicinal product	Herbal remedies (or herbal products for medicinal purposes): herbal products which are used for medicinal purposes but which are not regulated or authorised, including traditional herbal medicines.

EU Directive 2001/83/EC defines medicinal products as “any substance or combination of substances presented as having properties for treating or preventing disease in human beings” or “any substance or combination of substances which may be used in or administered to human beings either with a view to restoring, correcting or modifying physiological functions by exerting a pharmacological, immunological or metabolic action, or to making a medical diagnosis” (13). Then, EU Directive 2004/24/EC, which amends EU Directive 2001/83/EC, defined herbal medicinal products as “medicinal products that exclusively contain as active ingredients one or more herbal substances, or one or more herbal preparations, or one or more such herbal substances in combination with one or more such herbal preparations” (14). The 2004 Directive then goes on to define herbal substances as:

“*All mainly whole, fragmented or cut plants, plant parts, algae, fungi, lichen in an unprocessed, usually dried, form, but sometimes fresh. Certain exudates that have not been subjected to a specific treatment are also considered to be herbal substances. Herbal substances are precisely defined by the plant part used and the botanical name according to the binomial system (genus, species, variety and author)*” (14).

On the other hand, it defines herbal preparations in the following way:

“*Preparations obtained by subjecting herbal substances to treatments such as extraction, distillation, expression, fractionation, purification, concentration or fermentation. These include comminuted or powdered herbal substances, tinctures, extracts, essential oils, expressed juices and processed exudates*” (14).

## 3 EU regulation of herbal medicinal products

There are now three ways of putting a herbal medicinal product into the EU market: full marketing authorization (13); Well-Established Use herbals (WEU) (13); and Traditional Herbal Medicinal Products (THMP) (14).

The marketing authorisation of herbal medicinal products possible under Article 6 of Directive (13) is subject to the same requirements as other medicinal products as outlined in Article 8(3) of the same Directive. Applying for a marketing authorization in line with Article 8 is thus very expensive, involving high costs in development and registration, and is more difficult to attain for herbal medicinal products. The product documentation requires a complete dossier with quality documentation, including clinical trials, safety data showing a favourable benefit-risk ratio, toxicology testing, and pharmaceutical studies. All favourable and less favourable observations also need to be documented.

The amendment of Directive 2001/83/EC by Directive (14) was a significant step forward, since it now offered a new path—the THMP—which involves a simple registration and is less expensive. This requires that the THMP has thirty years of usage, fifteen of which are in the EU. Products registered in this way are available either as mono-products, containing one ingredient, or combinations containing several active substances. Minimizing the number of active substances present in herbal combinations, a practice called “minus variants”, facilitates the registration of the THMP. Table 2 summarizes the characteristics of an herbal medicinal product to be marketed as a THMP.

**Table 2:** Characteristics of an herbal medicinal product to be granted a marketing authorisation as a Traditional Herbal Medicinal Product (THMP).

Legal basis for marketing authorisation	Requirements
Directive 2004/24/EC 2004, art. 16 (a simplified registration procedure for “traditional-use registration”)	Criteria of the herbal medicinal product: Evidence that the herbal product had thirty years of usage, fifteen of which were in the EU. Efficacy evidence is not required, but the product should prove not to be harmful and that its efficacy is plausible. Products with an established Community Herbal Monograph for herbal medicinal products. General therapeutic claims. Indications exclusively appropriate to traditional herbal medicinal products. Intended for use for diagnosis and treatment without the supervision of a medical practitioner. Administered exclusively with a specified strength and posology. Oral, inhaled or external routes. Process of evaluation: Bibliographic review of safety data Pharmaceutical quality Manufacturing, analytic and stability data Application is to be submitted to the Competent Authority of the Member State

Herbal products may use another path for registration: Well-Established Use (WEU). Article 10 (1a) of Directive 2001/83/EC provides for “well established use”, whereby medicinal products which have been extensively used clinically within the European Union can be regularized using this classification. The herbal product manufacturer generates this data from a bibliography, including literature reviews and epidemiological studies. Any available documentation containing both favourable and unfavourable evidence should be included, and reference to other products containing the same ingredients is necessary for the WEU system. This method is more expensive than the THMP path, but the herbal product can have more substantial claims of therapeutic indications, thus increasing its marketing potential. Table 3 summarises the characteristics required of a herbal medicine to be able to be authorised as an herbal medicinal product through the WEU route.

**Table 3:** Characteristics for an herbal medicinal product to be granted a marketing authorisation for well established Use.

Legal basis for marketing authorisation	Requirements
Directive 2001/83/EC Article 10 (1)(a)(ii)	Criteria of the herbal medicinal product: Extensive clinical use and experience with the constitutent/s of the medicinal product. Products with an established Community Herbal Monograph for herbal medicinal products. Recognised efficacy and an acceptable level of safety are shown by a detailed scientific bibliography. No limits to therapeutic indications. Process of evaluation: Quality Documentation showing the quality of the product. No new data from clinical trials.

Directive 2003/63/EC (15) gives specific requirements required to handle the quality standards of herbal medicine in an application dossier. This additional information consists of providing information on the product, name, address and responsibilities of the supplier, the processes involved in the production, including storage and transport, the geographic source of the herbal preparation, batch analysis results, and analytic validations (16).

The European Medicines Agency (EMA) has been instrumental in setting up guidelines to enhance the safety assessment of herbal medicinal products (17). However, challenges persist as different EU countries often classify these products in varying ways, hindering the establishment of a unified system.

## 4 Ethical considerations in the use of herbal medicinal products

Speaking at the International Conference on Traditional Medicine for Southeast Asian countries which convened in February 2013, Dr Margaret Chan, at the time the WHO Director-General, argued that:

“*Traditional medicines, of proven quality, safety, and efficacy, contribute to the goal of ensuring that all people have access to care. For many millions of people, herbal medicines, traditional treatments, and traditional practitioners are the main source of health care, and sometimes the only source of care. This is care that is close to homes, accessible and affordable. It is also culturally acceptable and trusted by large numbers of people. The affordability of most traditional medicines makes them all the more attractive at a time of soaring healthcare costs and nearly universal austerity. Traditional medicine also stands out as a way of coping with the relentless rise of chronic non-communicable diseases*” (18).

The Director-General thus confirms that “of proven quality, safety and efficacy” are the main ethical aspects requiring regulatory attention. The regulation of herbal medicines can thus be compared to the process of introducing a new molecular medicine into the market. This means that the efficacy and safety of herbal medicine should be established through reliable scientific methods, such as clinical trials. These should also be monitored throughout the life cycle of the medicinal product via the pharmacovigilance processes. The availability of any medicinal product on the market without evidence to support its efficacy and safety is highly unethical as it may have serious deleterious consequences for patients. The greater the possible harm, the greater is the duty of health authorities to regulate and take action. These concerns will thus be discussed next.

### 4.1 Quality

The quality of the sources used to produce herbal medicines is a very important ethical issue as it affects their safety and efficacy. Plant source quality is determined by intrinsic and extrinsic factors. Intrinsic factors relate to the genetic makeup of the plant, which can vary even between different cultivars of the same plant (19). Extrinsic factors relate to environmental conditions, agriculture, cultivation, and storage. These variations in plant characteristics determine the active ingredients of the plant, making quality control of the raw material challenging (16). To ensure better quality, the WHO recommends implementing quality assurance and control measures, including Good Manufacturing Practice (20). These standards must be applied both to the finished product and to the active substance. A Qualified Person must be responsible for ensuring the quality of the herbal medicinal product (21).

The presence of contaminants is another serious issue concerning the quality and safety of herbal medicines. These contaminants may be either biological or chemical. Biological contamination typically consists of microbes, such as bacteria, moulds, yeasts, viruses, and nematodes. At the same time, chemical contaminants may include mycotoxins and aflatoxins, agrochemical residues such as pesticides, toxic heavy metals as well as arsenic, nitrites and nitrates, and even radioactive contaminants (22). These contaminants are responsible for various adverse reactions reported from the use of herbal medicines. An overview of systematic reviews revealed that these included agranulocytosis, meningitis, multi-organ failure, heavy metal poisoning, and carcinomas, among other serious consequences (23). The same systematic review concluded that contamination was most common in Indian and Chinese products.

Adulteration of herbal medicines is another potential threat to their safe use. Adulteration refers to the undeclared content in the medicine and may be due to replacing one botanic species with another. Adulteration may occur deliberately, for example, if the original plant is more expensive. A case in point involved the replacement of *Stephania tetrandra* by *Aristolochia fangchi* in a weight loss product in Belgium, which caused terminal renal failure in at least 30 patients (24). Adulteration of herbal medicines may also result from the deliberate addition of pharmaceutical drugs. For example, when analysing 150 supplements to improve sexual performance, Gilard and colleagues found that 61% of these supplements were adulterated with PDE-5 inhibitors, with 27% of the supplements being adulterated with sildenafil (the active drug of Viagra), tadalafil and vardenafil, and 34% with their structurally modified analogues. Moreover, 25% of these adulterated supplements had a dosage higher than the recommended maximum (25). Doctors in Hong Kong have reported a case of a 33-year-old with epilepsy who died due to phenytoin poisoning after taking a Chinese herbal product augmented with phenytoin (26). It is to be expected that such adulteration could cause severe harm since the therapeutic window of phenytoin is small.

The issue of quality, however, is also present in conventional medicines. Both the Organisation for Economic Co-operation and Development and the EU Intellectual Property Office have reported the counterfeiting of drugs, leading to severe adverse reactions and a lack of public trust in regulatory bodies (27). Things, however, have greatly improved due to strict regulations in export, import and control measures during transport and storage. The serialisation process has significantly reduced the falsification of medicines, even though there are still rare reports of falsified drugs. Even concerning herbal medicinal products, the Good Manufacturing Practice improved the quality of these products (28).

### 4.2 Safety

Many people believe that natural products, including herbal medicines, are safe. However, global reports of adverse reactions associated with these products belie this belief. Advertisements promoting the natural characteristics of herbal medicines have led many consumers to believe them to be safer than conventional medicines (29). As a result, many pharmacies and health shops now stock various herbal preparations, and consumers often use them for self-medication. Additionally, many vitamin preparations include herbal substances or preparations in their formulae, which further complicates matters of safety for healthcare professionals and regulatory authorities.

Herbal medicines can vary widely in content, potency, and quality. Certain jurisdictions apply similar regulatory efficacy and safety standards for herbal medicines and synthetic drugs whereas other countries apply little regulation concerning this issue. Hence, the parameters of safety and use cannot be guaranteed. Evidence shows that, at most, 10% of herbal products in the global market are standardised (30).

Lack of standardization can lead to variability in the efficacy and safety of herbal products. The active compounds in herbs can vary depending on plant species, growing conditions, harvesting methods, and processing techniques. This variability can make it difficult to predict the effects of herbal remedies and may lead to inconsistent results and safety issues. For example, when plants face adverse conditions such as severe drought, they enact a natural defence mechanism to counteract this adversity and, as a result, produce toxic secondary metabolites (31). Medicinally relevant plant species such as *Digitalis purpurea* and *Atropa belladonna* show this phenomenon (30). Once the plant produces these toxic metabolites, it is not easy to separate them from therapeutic substances.

There is also little to no information related to toxicity issues of herbal medicines. Toxicity tests, therefore, are important in order to guarantee the safety of these products for patients and consumers as these can identify toxic compounds and determine their levels of toxicity. Moreover, chemical procedures, such as reduction processes or chemical group alterations, can modify and reduce the harmfulness of these poisonous substances (32).

Reports of persons experiencing adverse reactions when administered herbal medicines are common. Many of these adverse reactions are minor complaints, but healthcare professionals report several severe adverse reactions and even deaths (33). Millions of Asians and others who consume herbals containing Aristolochic extracts are at risk of suffering from chronic kidney failure and cancer of the upper urothelial tract (34). Aristolochic acid, the toxic principle of Aristolochic species, has been detected in other human cancers, including hepatocarcinoma (35). A review of hepatotoxicity reported that more than a hundred herbals used in traditional Chinese medicine cause liver toxicity (36). Moreover, many commercialised herbal remedies in European and US markets are of poor quality and are linked to adverse reactions, with the FDA estimating that dietary supplements cause 50,000 adverse events annually (37). Furthermore, healthcare professionals witness drug-herb interactions as well as negative impacts on medical laboratory tests.

With the introduction of Directive 2004/24/EC since 2004, monographs were set for herbal medicinal products, addressing the above issues. In the European Union (EU), monographs play a crucial role in ensuring the safety and efficacy of herbal medicinal products. Monographs are detailed documents that provide standardised information on the quality, composition, and therapeutic uses of herbal substances or herbal medicinal products, which contribute to harmonising standards across EU member states. Specifications set by monographs ensure consistency in the quality of herbal raw materials used in manufacturing herbal medicines and outline analytical methods and quality control parameters. Monographs include safety data and information on potential risks associated with the use of herbal substances or herbal medicinal products and may include known adverse effects, contraindications, interactions with other medicines, and precautions for specific patient populations. Monographs also provide evidence-based information on the therapeutic efficacy of herbal substances or herbal medicinal products. Manufacturers of herbal medicines in the EU are required to demonstrate compliance with monograph specifications as part of the regulatory approval process. This ensures that herbal medicines on the market meet established standards for safety, quality, and efficacy (38).

Herbal medicines also lack safety data for certain vulnerable patients such as children, women and older persons, a concern which remains an issue even in (authorised) conventional medicine. Pregnant women need the assurance that herbal medicinal products have reproductive and development toxicity data in place. Most herbal products, including herbal medicines, lack evidence of safety during pregnancy. Many pregnant women, however, perceive herbal products to be natural and safe (39). A 2015 study conducted at Royal Aberdeen Maternity Hospital, northeast Scotland, reported that more than 60% of the respondents to the survey used complementary and alternative medicine, excluding vitamins and minerals, in the third semester of their pregnancy, with 38% of the respondents using herbal products (40 different products) (40). A multicentre study carried out by Fachinetti and colleagues reported that the consumption of herbals during pregnancy (especially almond oil spreading) had unfavourable effects on the gestation period and birth weight (41). Recommendations on the labels of herbal products should clearly state whether the product is safe during pregnancy and lactation. Monitoring internet sites selling herbal medicines by national health authorities is essential to check for any claims regarding these vulnerable populations.

Carcinogenic toxicity is a very important ethical issue that is of significant concern to consumers/patients and health authorities. Regulatory agencies take this issue of carcinogenicity seriously and require tests to guarantee the safety of patients. These tests are costly, and the R&D process for conventional drugs demands their documentation (42). Most herbal medicines, however, have never been tested for carcinogenicity. An excellent way to reveal the carcinogenic effects of herbal medicine would be through observational and epidemiological studies. These, however, are yet to be carried out for most herbal medicines. Some medicinal plants are in great demand, raising serious concerns. Ginkgo Biloba, for example, has been found to induce liver tumours while also targeting the thyroid and the nose in animals, and clinical studies have reported significantly increased instances of breast and colon cancer (43). Long-standing use of herbal medicine does not imply 100% safety (42).

Another potential toxicity of significant concern is genotoxicity. Some plants possess this dangerous potential (44). In 2006, EMA published guidelines to assess the limits of genotoxic impurities, revising them in 2017 and adopting them in 2018 (45). Determining the genotoxicity issue in herbal medications is a challenge, needing the identification of suitable methods to aid in better regulation and safety.

### 4.3 Pharmacovigilance

While the registered number of adverse reactions with herbal medicines is tiny compared to those associated with conventional medicine, there is always a risk of harm, and it is important to have an efficient pharmacovigilance system. Directive 2010/84/EU defines a pharmacovigilance system as:

“*A system used by the marketing authorization holder and by Member States to fulfil the tasks and responsibilities listed in Title IX and designed to monitor the safety of authorized medicinal products and detect any change to their risk-benefit balance*” (46).

A significant challenge for better guaranteeing the safety of herbal products is the difficulty in the pharmacovigilance process of these preparations compared to conventional medicines (47). A herbal medicinal plant may contain several natural constituents, unlike a conventional medicinal product with only one active ingredient. Moreover, there is a significant variation in the herbal species from different geographic locations, resulting in variations in the natural ingredients of each species (7). Another challenge in the pharmacovigilance system is the need for clarity about which naming system to use when referring to an herbal product. Some manufacturers use the botanical name while others use the pharmaceutical name or the herbal drug name, which indirectly leads to problems in validating the botanical identity of herbal ingredients (48).

Difficulties also exist, however, in the pharmacovigilance of conventional medicines. The effectiveness of post-marketing surveillance for conventional medication is very much in doubt because there is a relatively low number of reports of adverse drug reactions. Researchers in the UK estimated that less than 10% of all severe adverse drug reactions are reported in the UK, even though the UK was one of the most significant contributors to pharmacovigilance (49), but today the UK no longer participates in the EU centralized reporting system. Hence, most adverse drug reactions arising from the use of conventional medicine are unreported. This situation opens a Pandora's box of questions. How can a patient on conventional medicine be fully informed about the possible adverse drug reactions when the incidence of such reactions is very approximate? Millions of individuals from all corners of the world have used herbal remedies for centuries. Do these historical herbs need pharmacovigilance after being in use for centuries? At the same time, however, even though they have been used for centuries, their toxicity (throughout the ages) has yet to be established.

### 4.4 Efficacy

One of the main concerns about the use of herbal medicines is the lack of scientific evidence regarding their efficacy. Herbal medicines are often viewed negatively because they are thought to prevent, hinder or delay access to care based on conventional medicine. The refusal of the latter may stem from a person's belief that herbal medicine is superior to conventional treatment. However, this delay in using evidence-based treatment can have serious consequences. For instance, children are at risk when their parents insist on using only herbal medicines even if their physicians advise conventional treatment. In 2010, four children reportedly died in Australia due to their parents' refusal to use conventional treatments, insisting on using herbal medicines only instead (50).

Even though herbal medicines have been used for centuries—based on traditional knowledge and anecdotal evidence—there is a growing interest in studying their efficacy through scientific research. However, the results of such studies have been mixed. While some herbal medicines have shown therapeutic potential and are supported by scientific evidence, others have not demonstrated significant benefits in controlled clinical trials. For example, St John's Wort has been extensively studied for its efficacy in managing mild to moderate depression, and it has shown positive results in some trials. Nonetheless, more research is needed to determine the efficacy of other herbal medicines (51). Similarly, though ginseng is not as deeply ingrained in European culture as in Asia, its use in various sectors like health supplements, food and beverage, and cosmetics is notable (52). In recent years, the WHO has approved ginseng as an adaptogen and tonic and an anti-stress and anti-fatigue agent (53). Clinical trials with ginseng included improving sexual and cognitive functions. Yet, despite many trials, the medicinal properties of ginseng remain inconclusive (54). An extensive overview of ginseng clinical trials from 1979 to 2018 by Chen and colleagues concluded that despite its global popularity, the clinical efficacy in various medical conditions remains to be verified (55). The Committee on Herbal Medicinal Products (HMPC) arrived at the same conclusions as these researchers, concluding that based on ginseng's long history, it is indicated for tiredness and weakness (56).

The Evidence-Based Medicine Working Group initiated the paradigm shift that changed the practice of medicine (57). This initiative resulted in the philosophy that all healthcare interventions ought to be rooted in a scientific evidence base. The historical evidence of herbal medicine use does not impress evidence-based scientific supporters. Random Controlled Studies (RCTs) and systematic reviews, with or without meta-analysis, stand at the top of this hierarchy of evidence.

Researchers trained in biomedical and clinical research methods argue that RCTs are the only valid source of knowledge regarding clinical efficacy (58). Herbal medicine researchers, however, reject the argument that only RCTs generate knowledge, considering this attitude to be Western imperialistic reasoning (59). They blame the arrogance of Western researchers who expect traditional herbal medicine to be assessed using biomedical technologies and vocabulary. These diverging opinions lead to an important question. While it is undoubtedly the case that RCTs are known to develop knowledge—even though this is generated from a limited number of patients over a limited period—and that knowledge creation has a vital social value, does the experience of millions of people using herbal medicine for centuries have no significant efficacy and social value?

Different approaches in research methodology can lead to misunderstandings and conflicts when evaluating the efficacy of herbal medicines. American researchers, for example, may want to test the efficacy of a herbal medicine on chronic fatigue syndrome. To do so, they would use the Western medical classification system to determine the inclusion and exclusion criteria. This classification, however, would be very different from the perspective of Chinese traditional herbal medicine which is based on the belief that pathogenesis is to be interpreted with the help of Qi, the vital force forming part of any living entity, and Yin and Yang, the basic forces in the universe (59). Western healthcare professionals may dismiss this theory as unscientific, but a different approach could be helpful. For instance, researchers have focused on patient-reported outcomes and developed a new health-related quality-of-life instrument based on Chinese culture and medicine (60). Traditional herbal medicines that increase Yang may enhance mitochondrial-driven biological activity in the human body and benefit patients suffering from chronic fatigue syndrome. Therefore, the concept of Yang deficiency in traditional herbal medicine can be compared to the aetiology of chronic fatigue syndrome (61).

The same discourse occurs when considering a study's total outcome measures. The SF-36 quality of life instrument measures physical and physiological well-being (62), but this instrument is not suitable within the terminology and ideas of traditional herbal medicines. Hence, investigators need to construct and validate analogous measures that more faithfully detect the effects of herbal medicine.

International medicines regulatory authorities, such as EMA, impose pre-market testing of new drugs before they are approved with marketing authorization. There is increasing pressure to provide this kind of evidence to prove the efficacy of herbal medicines for them to be authorised as herbal medicinal products. Since pharmaceutical companies spend millions of euros to introduce any new drug globally, manufacturers must invest heavily in research and development if they seek essential therapeutic claims for their herbal medicines. However, while regulatory authorities demand RCTs and systematic reviews to prove the efficacy of medicines, evidence shows that even in the case of conventional medicine, efficacy is not as strong as usually believed (63). Evidence-based medicine, for example, works best for single diseases and not for patients with complex comorbidity, frailty, and dementia, and yet the exclusion of older persons with comorbidities from clinical trials “means that much of the evidence isn't fit for purpose” (64). A study in 2017 found that 51% of recommendations in the US primary care only had good or fair evidence based on patient-oriented evidence. Only 18% were based on high-quality evidence based on patient-oriented evidence from consistent, high-quality studies (65). It is reasonable therefore to ask whether there is a bias against herbal medicine studies: whereas reasonable evidence of efficacy is acceptable in the case of conventional medicine, clinical researchers continue to insist on higher evidence for herbal medicine.

Furthermore, conditional marketing authorization is a critical tool in the EU's regulatory arsenal, allowing for faster patient access to essential new medicines while ensuring that safety and efficacy standards are met through rigorous post-approval commitments (66). Similar expedited pathways exist in other regulatory jurisdictions, like the FDA's Accelerated Approval in the United States, which also allows for earlier approval of drugs based on surrogate endpoints that are thought to predict clinical benefit (67). Currently, there is an increased use of real-world data after marketing authorisation to support the continued authorisation of medicinal products with a conditional marketing authorisation. In comparison, the regulatory framework for traditional herbal medicinal products ensures that the evidence of use of these products is available before, rather than after, marketing authorisation. Thus, the model for herbal medicinal products actually may offer a better standard for efficacy for medicinal products.

### 4.5 Environmental sustainability

Society is increasingly becoming aware of the importance of sustainable development, and this also holds true for the herbal medicine market. The pursuit of profits can put a lot of pressure on certain high-demand plant species, which can have a negative impact on the environment. It is important for society to be mindful of the environment and to cultivate a balanced human-environment relationship to promote the health and well-being of citizens. When certain medicinal plants are in high demand, manufacturers may focus on producing these plants to maximize profits, which can lead to the near extinction of many other valuable medicinal plant species worldwide. This situation can pose a threat to biodiversity and should be a cause for concern (68).

Biodiversity is essential for traditional medicine systems worldwide as it guarantees a wide range of plants with medicinal properties. Overharvesting can lead to the depletion of certain plant species, affecting availability and ecosystem health (69). Hence, it is crucial to use sustainable and responsible cultivation and harvesting practices to preserve biodiversity (70). The survival of medicinal plants and the people who depend on them is threatened due to unsustainable harvesting practices. This not only affects individuals' livelihoods but also erodes cultural knowledge and the potential for discovering new molecules. Additionally, there is a concern about biopiracy, as traditional herbal medicines are often patented without the original owner's consent or compensation (29).

While biodiversity *per se* is not usually part of the regulatory concerns, the EMA is already moving towards environmental risk assessment of medicinal products for human use, including herbal medicinal products, if applicable (71).

## 5 Competing perspectives of stakeholders of the herbal medicine market

There are various stakeholders who have an interest in the herbal medicine market: users of these remedies, researchers in herbal medicine, manufacturers of these products, practitioners of both herbal and conventional medicine, and health regulators. Each stakeholder might focus on different issues and prioritise different options, such as insisting on safety, quality, and efficacy, or arguing for equity, access and affordability. Thomas M. Jones' moral intensity model may be useful in this context. Defined as “the extent of issue-related moral imperative in a situation” (72), moral intensity includes several dimensions that could influence moral awareness (or sensitivity), judgement, intention and behaviour, the four stages of ethical decision making identified by James Rest (73). A crucial factor in ethical dilemmas, therefore, is the significance of the issue to the stakeholders, and this determines their ethical response. Stakeholders who prioritize their self-interest, profits or personal gains, or their immediate satisfaction may suffer from ethical myopia, where they have a narrow view of the ethical dilemma and thus focus only on short-term benefits, ignoring the long-term consequences and negative impacts (74). These stakeholders may also exhibit ethical fading, where ethical concerns are gradually pushed into the background, leading to a slow but steady shift towards self-interested goals (75). Over time, ethical fading and ethical myopia may become normalized behaviour for stakeholders. For example, a corporation specialising in the marketing of herbal medicinal products may engage in online advertising that includes erroneous information regarding the efficacy of their products. Over time, this practice worsens as ethical considerations become less significant, and the company continues advertising deceptive information.

In many parts of the world, including some European regions, traditional herbal remedies remain a significant aspect of healthcare, especially in areas where access to conventional healthcare is limited or where traditional practices are deeply rooted in the culture. Affordability and respect for one's traditions are the primary objectives of these users. Efficacy, safety, and quality factors have a lower subjective importance. These traditional practices are passed down from generation to generation, and these populations believe in their effectiveness in treating various health conditions. Demanding too strict regulations for these populations could cause pathogenic vulnerability. This vulnerability arises when an attempt to regulate has the paradoxical effect of exacerbating existing vulnerabilities or creating new ones (76). Burdensome regulations can leave these people without affordable medical care.

At the same time, prioritising price and tradition over the quality of herbal medicines, could have harmful consequences. This is especially true for low-income individuals who may have budget constraints and limited options, making them more susceptible to purchasing low-quality or adulterated herbal remedies. This puts their well-being at risk, and they may need accurate information about the potential benefits and risks of such products. These individuals may also be vulnerable to unscrupulous herbal practitioners or sellers who exploit their economic circumstances and lack of knowledge. Additionally, when these individuals encounter medically-trained physicians who do not have a high opinion of herbal medicines, they may feel stigmatized and experience moral distress (77).

While for low-income EU citizens, distributive justice and a lack of respect towards their traditions are the ethical issues regarding access to herbal medicines, for higher-income citizens, the main ethical challenge is autonomy. The liberal tradition places great emphasis on the moral and political significance of rational self-rule, in particular, the freedom from the interference of others in one's choices and decisions, even if, for a knowledgeable professional like the physician, these decisions appear unreasonable. Physicians are not experts in spirituality, personal worldviews or the various situations unique to a patient. Hence, patients can make risky decisions because they know precisely the fine details of their context. Patients may, for example, choose an herbal product because their idea of health is to be as close as possible to nature. Moreover, for users of herbal medicines, the freedom to self-medicate signifies a personal gain in autonomous decision-making. However, this autonomous reasoning may have negative aspects since delaying or avoiding necessary medical care can worsen their health outcomes. Studies also indicate that herbal medicines are used concomitantly with conventional medicine, often without informing one's physician, and this can cause harm (78).

Allocation of funding is the primary ethical concern for herbal medicine researchers. Such financing is scarce and insignifant compared to the funding for research and development for conventional medicine. Some researchers from Western medical areas argue that funding for herbal medicine wastes resources (79), whereas other scientists argue that the latest technological tools can yield better and faster results in delivering new molecular entities to fight against complex diseases. Establishing evidence-based research to ensure safety, quality, therapeutic and clinical evidence crucial for the globalization of traditional medicine is very costly and comes at a high price. Herbal medicines could become prohibitively expensive for a large number of individuals.

Another stakeholder involved in the herbal medicine market is the herbalist. Regulation of herbalists in the EU varies between member states. Herbal practitioners, however, should be medically trained to advise patients regarding health matters. Unlicensed herbalists may recommend the use of herbal medicines to their patients without having accurate information on their effectiveness and safety. They may also make recommendations that interfere with physicians' advice about conventional treatments and immunisation (80). The most cited argument against these practitioners is that they may delay access to evidence-based medicines, and this might have serious consequences. There are, however, herbalists who take their role seriously and do their best to give honest advice to their customers. These herbalists would welcome more regulation and governance so that dishonest herbalists would discontinue their practice and remove any distrust associated with herbal medicine practitioners.

Healthcare professionals, such as physicians and pharmacists, may encounter ethical challenges related to the use of herbal medicines. While they are trained to provide evidence-based medical care in the best interest of the patient, a patient may want to use herbal medicines, creating a complex ethical dilemma. In such cases, the principles of beneficence and non-maleficence may collide with the principle of autonomy. Healthcare professionals, therefore, need to be more familiar with the safety, effectiveness, and mode of action of herbal medicines. However, many healthcare professionals have an inherent disbelief in the safety and efficacy of herbal medicine, which may affect their judgments. This may lead to tension in the healthcare professional-patient relationship and possible mistrust. Healthcare professionals need to inform patients about the benefit-risk ratio of herbal medicines. Since they are not familiar with herbal medicines, and most probably might not trust them, making medical decisions in these unfamiliar realms may make them uncomfortable.

## 6 Discussion

In Europe, the use of herbal medicines is on the increase. EU regulators have become morally aware that they are faced with a situation requiring a decision or action that could affect the interests, welfare, or expectations of many stakeholders in this herbal medicine market, including customers and society. Moral awareness, or sensitivity, involves identifying the ethically salient aspects of a situation and the various people involved to formulate possible courses of action that take into account the needs of various stakeholders (81). This moral awareness allows regulators to think about and imagine possible ways of finding the right balance between the claims of multiple stakeholders mentioned above, which can be summarised as quality, safety, and efficacy, on the one hand, and equity, access and affordability, on the other. Moral imagination helps health regulators to explore and understand ethical dilemmas beyond their present experiences (82). This element of moral awareness broadens the regulators' ethical horizons and promotes positive change and ethical progress. EU regulators give value to traditional herbal medicine, motivating European companies to expand their trading activities to a global scale in order to make healthcare more available and accessible. The greater the medicinal claims a product makes, the more its marketing power and its potential financial return. EU regulations provide manufacturers with three pathways to market their products in the EU territory with different requirements of evidence proportionate to the severity of the disease. The THMP pathway can be used by manufacturers to treat minor health conditions, which respects the cultural and traditional values of consumers who prefer natural remedies. However, if manufacturers want to market their products for moderate to severe diseases, they must provide scientific evidence to support their claims. The EU authorities prioritize public safety and aim to provide reasonable hope for patients' well-being.

The EU regulatory body's decision to provide three possibilities for manufacturers to place herbal medicinal products on the market of national EU territory may reflect the EU's position on two philosophical paradigms: the positivist and the interpretivist worldviews which underlie the different ontological and epistemological approaches of Western medicine and herbal medicine. These two philosophical worldviews differ in their views of reality and knowledge. Positivism exhibits a realistic ontology where only one reality exists, independent of personal views (83). Empirical epistemology can discover this reality. Knowledge comes from objective observation and testing, free from personal beliefs or interpretations. Positivism emphasizes the importance of empirical evidence and verification through experimentation and clinical trials. It thus aims to understand cause-effect relationships using scientific and deductive approaches, directing its focus on the standardization and replicability of scientific research. Positivism significantly influences the methodology and practices of Western medicine (84, 85), which uses protocols and clinical practice guidelines to diagnose and treat patients to minimize subjective influences. Regulatory bodies for health take this positivist view when granting marketing authorization for herbal medicinal products indicated for moderate to severe diseases.

Unlike Western medicine, traditional herbal medicine finds its roots in interpretivism. The interpretivist paradigm allows researchers to explore and evaluate experiences and perspectives of a particular social context (86). This philosophical worldview focuses on the subjective living experiences essential to understanding human behaviour during sickness. Interpretivism exhibits an idealist ontology, a multiple reality constructed through the individual's perceptions, experiences and contextual factors (83). Hence, interpretivism shows a subjective epistemology and quantitative data is of little interest (87). Herbal medicine originates in traditional histories and cultures, each having its own interpretations of the use of herbs in treating ailments. The effectiveness of a herbal remedy depends on the user's subjective beliefs and experiences, which may vary from one person to another. Herbal medicine views health and disease holistically, a view in line with the interpretivism philosophy of appreciating the importance of multiple realities in understanding the healing powers of herbs. Herbal medicine has a patient-centred approach where healing is tailored to the individual's unique conditions and context.

EU regulators give voice to both paradigms. Whereas the EU supports the positivist paradigm when deciding whether to license a product for severe disease, it supports the interpretivist approach to registering herbal products for minor ailments. Any regulatory or political body must find an ethical compromise in its decisions in order to balance the different claims of various stakeholders. In order to do so, they need to consider the contrasting views of duty-based ethics and consequentialism. Proponents of duty-based ethics may argue that certain moral principles, such as truth-telling (including claims to safety, efficacy and quality) and informed consent (with an emphasis on truthful information) should be upheld as non-negotiable duties by healthcare professionals and regulatory bodies. They may advocate for strict regulations prioritizing consumers' safety and well-being, insisting on rigorous testing, labelling, and quality control, even at the risk of limiting access to certain herbal products. Consequentialists might argue that the benefits of increased access to herbal remedies, which may be more affordable or culturally important to some, may outweigh the potential risks. They may thus support a more permissive regulatory approach that allows consumers more freedom of choice, even if this means accepting some risks associated with herbal medicines. Duty-based ethics would thus tend to lean towards more stringent regulations to protect consumers, while consequentialist ethics would prioritize individual access. Another clash occurs when balancing the allocation of resources. Whereas deontologists insist on strict regulation, consequentialists argue that stringent regulations on herbal medicines move financial resources away from more basic healthcare needs, eventually leading to a less efficient public health service. Another tension exists regarding cultural and ethical pluralism. Consequentialists advocate respecting cultural diversity and individual preferences, suggesting that such behaviour enriches societal values. Duty-based ethics, however, emphasizes universal principles that may sometimes conflict with certain cultural practices. Another tension arises concerning informed consent. Duty-based ethics demands comprehensive labelling and information dissemination of herbal medicines to ensure that all consumers are fully informed about the benefits and risks of these products. Consequentialists believe that excessive information would scare away individuals from using herbal medicines and thus deprive millions of persons of their only affordable and accessible healthcare. The EU triple pathway for regulating herbal medicines thus finds an ethical balance between these claims.

EU regulators of herbal medicine have done an excellent job. More work, however, is still needed to make sure that the right to health is really a universal right. EU and global policymakers must consider national, historical, cultural, and religious backgrounds. The principle of fairness acknowledges a constellation of knowledge in implementing plural healing and plural legal systems without seeking to valorize one knowledge over another (88). Western philosophers should not reject all lessons coming from non-Western origins. The WHO Traditional Medicine Strategy 2014–2023 resonates with Sustainable Development Goal 3, ensuring healthy lives and promoting well-being for all ages and aims to reach this balance (18). Integrating traditional and conventional medicine can contribute to the long-sought ambition to achieve universal health coverage, address health emergencies and promote global well-being.

In its Global Report on Traditional and Complimentary Medicine 2019, the WHO examined the European region (the WHO European region, which is wider than the EU and the Council of Europe) to evaluate whether it would be reaching the WHO's strategic goals (89). This report commented that there was a marked increase in the number of states with a registration system and regulation for herbal medicines, with 45 of the 53 states reporting having both (89). However, indicators such as national policies, offices, programs, research initiatives, and Traditional and Complementary Medicine lag significantly behind global averages. Only 36% of countries in the WHO European region have a national policy on traditional medicine, 40% have a regulatory framework on traditional medicine, 28% have a national office for traditional medicine, 36% have an expert committee specialised in traditional products and therapies, and only 36% have a research institute on this traditional branch of medicine (89). These statistics indicate that more must be done to reach the WHO Traditional Medicine Strategy 2014–2023 goals, and that even in the EU, which has a regulatory framework, more needs to be done to encourage the increased use of herbal medicinal products.

At the same time, EU regulators, both at the EU and national level, must be more active in preventing online misinformation about herbal remedies. Today, the internet is the primary source of information for most EU citizens and it is thus easy to assume that most EU citizens use it to obtain information on herbal medicines. EU policymakers must also insist on education related to herbal medicine for Western healthcare professionals. Education and access to unbiased information on herbal medicine are essential in delivering care to patients during their choice of medicines. Healthcare professionals must keep abreast of scientific developments in herbal medicine research to improve their knowledge and expertise. This openness to learning from a different medical source helps healthcare professionals be in a better position to deliver patient-centred care, allowing better communication and fostering increased trust between professionals and their patients. This is particularly true of pharmacists. A study in 2007 indicated that consumers prefer to seek information about herbal medicine from pharmacists (90). Curmi reports that 65% of the respondents in his study seek advice on herbal medicines from pharmacists, 19% from doctors and 16% from health shop employees (52). Most consumers seek information regarding adverse reactions, interactions with conventional medicines and contraindications. All these questions require the pharmacist to be well-trained in herbal medicine and have access to the necessary information when needed. Curmi reports that pharmacists attained a mean knowledge score of 27 out of 56 in a validated questionnaire study. At the same time, health shop employees obtained a mean knowledge score of 28 out of a maximum score of 56 (52). These results indicate that pharmacists and health shop assistants need better knowledge of herbal medicines. As a leading healthcare professional, this situation puts great responsibility on the pharmacist to remedy this lack of expertise as soon as possible.

## 7 Conclusion

There are now twenty years of experience with the EU regulatory framework for herbal medicinal products as implemented by the update of Directive 2001/83 through the introduction of Directive 2004/24/EC. This framework sought to achieve a synergistic effect between the regulation and the use of herbal medicines for treatment. This framework has been quite successful in achieving a balance between safeguarding the basic principles of regulation of medicinal products, mainly quality, safety and efficacy, while at the same time supporting the principles for the use of medicines including equity, access and affordability of medicines for citizens. This regulatory framework attempted to find a fair balance between stakeholders' claims. It enabled regulators to safeguard the well-being of citizens while allowing citizens to participate in their choice of treatment, in collaboration with healthcare professionals as needed. Industrial stakeholders were free to engage in the market and the principle of free movement of goods was protected. It established a robust structure to ensure the standardisation of scientific data and streamline the monitoring procedure for these items. This EU framework could therefore serve as a practical guidance for the use and regulation of herbal medicines, even outside the EU.

The lessons learnt from the EU regulation for herbal medicines could also perhaps be adopted and adapted for the fair regulation of other products within the EU market. Interestingly, the EU regulatory framework for medical devices has been recently revised, even though unfortunately there has been a lack of infrastructure for its implementation, leading to bottlenecks. The regulatory framework for substances of human origin (SOHO) has also been approved recently. Hopefully, this regulation will not impede access to such substances as, in fact, the hospital exemption intends to do. Presently, the EU legislation for medicinal products is also being revised. While this is currently considered to be the most stringent regulatory framework to safeguard safety and efficacy, in reality, the principle of conditional marketing authorisation, introduced into this legislative framework in a previous revision, has shifted the accumulation of evidence of efficacy and safety to the post-authorisation phase, through the use of real-world data. In practice, therefore, traditional herbal medicinal products seem to have a stronger regulatory framework in terms of efficacy and safety than products authorized through conditional marketing authorization. The same moral imagination and courage shown by regulators in the case of herbal medicines could perhaps be used in the regulatory frameworks of other healthcare products.
